# Molecular Cloning and Immunochemical Characterization of a New Japanese Cedar Pollen Allergen Homologous to Plant Subtilisin-Like Serine Protease

**DOI:** 10.1097/WOX.0b013e318201d81d

**Published:** 2010-11-15

**Authors:** Ahmed Ragaa Nour Ibrahim, Seiji Kawamoto, Keisuke Mizuno, Yayoi Shimada, Satoshi Rikimaru, Nobukazu Onishi, Kunihiko Hashimoto, Tsunehiro Aki, Takaharu Hayashi, Kazuhisa Ono

**Affiliations:** 1 Department of Molecular Biotechnology, Graduate School of Advanced Sciences of Matter, Hiroshima University, Higashi-Hiroshima, Japan; 2 JST Innovation Plaza Hiroshima, Higashi-Hiroshima, Japan; 3 Nishikawa Rubber Co. Ltd., Hiroshima, Japan; 4 Takanobashi Central Hospital, Hiroshima, Japan

**Keywords:** allergen, Japanese cedar pollen, serine protease, subtilisin

## Abstract

Protease activities in allergen sources are thought to be involved in triggering allergic inflammation through the disruption of epithelial barrier or the induction of proinflammatory cytokines. Protease allergens may also work as type 2 helper T cell (T_H_2) adjuvants through the cleavage of cell surface receptors. Here, we report molecular cloning and immunochemical characterization of a new Japanese cedar (*Cryptomeria japonica*) pollen allergen (CPA9) homologous to serine protease, which is initially found as a high IgE-binding spot on our two-dimensional (2-D) IgE immunoblotting map. The *cpa9 *cDNA encoded a 757 amino acid polypeptide showing a significant sequence identity with plant subtilisin-like serine protease family members including melon major allergen Cuc m 1. We found that native CPA9 purified from *C. japonica *pollen showed a high IgE-binding frequency and IgE cross-reactivity with melon extract.

## 

Japanese cedar (*Cryptomeria japonica*) pollinosis is one of the most serious allergic diseases in Japan, [1] and elucidation of its major allergen repertoire is critical for the development of component-resolved diagnosis and specific immunotherapy. Previous studies have exclusively been focused on the 2 major allergens Cry j 1 [2,3] and Cry j 2, [4,5] but the other allergen molecules have yet to be characterized thus far. Our group has reported the existence of 131 *C. japonica *pollen allergen spots on the 2-D IgE immunoblotting map, in which 31 spots shows higher IgE-binding frequency than that of Cry j 2 [6]. We also have identified additional new *C. japonica *pollen allergen molecules structurally belonging to isoflavone reductase (IFR) [7], class IV chitinase, [8] and nonspecific lipid transfer protein (LTP) [9]. The IFR allergen (CJP-6) is homologous to birch pollen allergen Bet v 5 [10] and pear allergen Pyr c 5 (GenBank Accession No. AF071477), and shows 76% (19/25) IgE-binding frequency [7]. The chitinase allergen (CJP-4) is a potent major allergen with 100% (31/31) IgE-binding frequency and IgE-cross-reactivity with latex C serum [8]. The LTP allergen (CJP-8) also cross-reacts with other LTP allergens from peach (Pru p 3) and *Parietaria judaica *pollen (Par j 1) [9]. Fujimura et al have recently reported a taumatin-like *C. japonica *pollen allergen Cry j 3 [11] with low IgE-binding capacity (27%, 27/100).

Many aeroallergens have been shown to possess proteolytic activity. The best-characterized protease allergen is the cysteine protease antigens Der f 1 and Der p 1 from the house dust mite (*Dermatophagoides farinae *and *D. pteronyssinus*). Proteolytic activity of those allergens may trigger proallergic inflammation through the proteolytic degradation of epithelial tight junctions, [12] the release of proinflammatory cytokines from mucosal epithelial cells, [13] or augmentation of IgE synthesis [14-16]. These cysteine protease allergens may act as a T_H_2 adjuvant through proteolytic cleavage of CD25 on T cells [17] or in concert with basophils [18]. Serine proteases represent additional major aeroallergens. The house dust mite contains 3 classes of serine proteases designated as the group 3 (trypsin), the group 6 (chymotrypsin), and the group 9 (collagenase) [19] allergens. Sun et al have shown that both Der p 3 and Der p 9 from *D. pteronyssinus *trigger the release of GM-CSF and eotaxin from human lung epithelial cells [20]. Indoor airborne allergenic fungi *Penicillium *species also contain the group 13 (alkaline serine protease) and the group 18 (vacuolar serine protease) family members, [21] and the *P. chrysogenum *Pen ch 13 allergen induces proinflammatory mediator releasing and degradation of a tight junction protein (occludin) in lung epithelial cells [22]. Allergenic pollen grains also contain a significant amount of proteases, [23] and indeed we have recently identified the first major *C. japonica *pollen allergen (CPA63) homologous to the plant atypical aspartic protease/nucleoid DNA-binding protein family members [24]. In this study, we describe cDNA cloning and immunochemical characterization of a new subtilisin-like serine protease allergen (CPA9), which has originally been found as a major allergen spot with a high IgE-binding frequency (50%, 20/40) on our 2-D IgE immunoblotting map [6].

## Materials and methods

### Human Sera

Sera from *C. japonica *pollen-allergic patients were obtained from 26 donors selected on the basis of clinical history and positive IgE-binding to *C. japonica *pollen (RAST score ≥2). Nonallergic sera from 5 healthy volunteers were used as a negative control. These sera were stored at -30°C until use. All experiments were approved by the Institutional Review Board of Takanobashi Central Hospital and were described in detail to all participants before they provided their informed consent and were admitted into the study.

### Time-of-Flight Mass Spectrometry (TOF-MS)

Our previous 2-D IgE immunoblotting study of *C. japonica *pollen [6] revealed that the allergen spot No. 9 (CPA9) was one of unidentified major allergens with high IgE-binding frequency (50%, 20/40). To obtain its partial amino acid sequence information, the CPA9 spot was excised from the 2-D SDS-PAGE gel, and subjected to tryptic in-gel digestion as described [6]. The MALDI-TOF-MS and tandem MS/MS analyses were then performed using an Ultraflex TOF/TOF spectrometer (Bruker Daltonics, Billerica, MA). The obtained MS and MS/MS spectra were submitted to Mascot database (Matrix Science, London, UK) for homology search. De novo peptide sequencing was carried out using BioTools software (Bruker Daltonics) and derived sequences were also subjected to Mascot (Matrix Science) for assignment of protein identity.

### cDNA Cloning

The extraction of *C. japonica *pollen RNA and cDNA cloning were performed as previously descried [24]. In brief, total RNA was extracted from frozen *C. japonica *anther (5 g, crashed in liquid nitrogen) using Concert Plant RNA Reagent (Invitrogen, Carlsbad, CA), and mRNA was selected by Oligotex-dT30 Super mRNA Purification Kit (Takara Bio, Otsu, Japan). cDNA templates for Rapid Amplification of cDNA Ends (RACE)-PCR were synthesized using BD SMART RACE cDNA Amplification Kit (BD Biosciences, San Jose, CA). 5'- and 3'-RACE PCR were then carried out using BD Advantage 2 PCR Kit (BD Biosciences) with *cpa9 *gene-specific primers (sequence information will be appeared elsewhere). Resulting PCR fragments were subcloned into pGEM-T Easy (Promega, Madison, WI), and both strands of the nucleotide sequences were determined using ABI PRISM 3100 *Avant *Genetic Analyzer (Applied Biosystems, Foster City, CA).

### Preparation of Recombinant CPA9 (r-CPA9) and Its Specific Antisera

Expression system of r-CPA9 was developed using *Escherichia coli *cold shock expression vector pCold-TF DNA (Takara Bio) as described [9]. Briefly, the full-length *cpa9 *cDNA was inserted into pCold-TF, and *E. coli *Rosetta gami was transformed with the resultant expression construct. Expression of trigger factor (TF)-tagged r-CPA9 (r-TF-CPA9) was induced upon cultivation of the transformant at 15°C, and the recombinant protein was then semi-purified on a HisTrap HP metal chelate affinity column (GE Healthcare Bio-Sciences, Uppsala, Sweden). The purified protein was then digested with thrombin (Novagen, Madison, WI) to remove TF-tag, and r-CPA9 was finally purified by SDS-PAGE and gel-extraction procedures as described [9]. To obtain CPA9-specific antisera, BALB/c mice (4 weeks of age, Charles River Japan, Yokohama) were immunized intraperitoneally with purified r-CPA9 using Titer-Max Gold adjuvant (CytRx Corporation, Norcross, GA) every 2 weeks for 8 weeks. The antisera were collected 10 days after the final immunization.

### SDS-PAGE and Immunoblotting

Protein samples were analyzed by SDS-PAGE on a 12.5% slab gel, and protein bands were detected by silver staining. For immunoblotting analysis of CPA9, protein samples were electronically transferred onto a polyvinylidene difluoride (PVDF) membrane (Immobilon-P, Millipore, Bedford, MA). After blocking with phosphate-buffered saline (PBS) supplemented with 0.05% Tween 20 and 5% skim milk, the blot was probed with anti-CPA9 sera (1:5,000 in the blocking buffer). After washing, the blot was reacted with secondary horseradish peroxidase-conjugated rabbit antimouse IgG polyclonal antibody (Sigma, St. Louis, MO). The protein bands were visualized on an x-ray film (Konica Minolta, Tokyo, Japan) using an ECL Plus Western Blotting Detection Kit (GE Healthcare Bio-Sciences). To detect His-tagged r-CPA9, the immunoblotting procedure with anti-His tag monoclonal antibody (GE Healthcare Bio-Sciences) was carried out as described [9].

### Purification of Native CPA9 (n-CPA9)

Crude *C. japonica *pollen extract was prepared as described [7]. The extract (50 g) was then dissolved in 1.5 M (NH_4_)_2_SO_4_/20 mM Tris-HCl (pH 8.6), and applied onto a HiTrap Butyl HP hydrophobic interaction column (GE Healthcare Bio-Sciences). The proteins were fractionated by decreasing the (NH_4_)_2_SO_4 _concentration. The active fractions containing n-CPA9 (assessed by anti-CPA9 immunoblotting) were concentrated/desalted, and then applied onto a Mono Q anion exchange column (GE Healthcare Bio-Sciences) to obtain n-CPA9. Identity of n-CPA9 sample was verified by the MALDI-TOF-MS and tandem MS/MS sequencing analyses as described above.

### Enzyme-Linked Immunosorbent Assay (ELISA) and Competitive ELISA

ELISA was used to evaluate the IgE-binding frequency of n-CPA9 as essentially described [7] with minor modifications. In brief, a 96-well Microtiter plate (NUNC-Immuno Plate Maxisorp F96, Nalge Nunc International, Roskilde, Denmark) was coated with 50 *μ*L of antigen solution (500 ng/ml in 100 mM bicarbonate buffer, pH 9.4) and incubated at room temperature for 2 hours. Then the plate wells were blocked with a buffer (3% skim milk/1% bovine serum albumin/PBS) overnight at 4°C. Subsequently, 50 *μ*L of diluted cedar pollinosis patients' sera (1:20, diluted with blocking buffer) was supplemented into the well, and incubated overnight at 4°C. Then 50 *μ*L of biotinylated goat antihuman IgE (1:10,000, Biosource International, Camarillo, CA) was added and incubated for 2 hours at room temperature, followed by incubation with 50 *μ*L alkaline phosphatase-conjugated streptavidin (1:10,000, Jackson ImmunoResearch Laboratories Inc., West Grove, PA) for 1 hour. For enzyme reaction, AttoPhos substrate solution (Promega) was added and incubated at room temperature for 30 minutes. Then fluorescence intensity of each sample well was analyzed using a Wallac 1420 ARVOsx Multilabel Counter (Perkin Elmer Life Sciences, Boston, MA). The cut-off value was defined as mean ELISA value + 3 × SD from normal healthy control sera (n = 5).

For competitive ELISA, CPA9-positive Japanese cedar pollinosis patients' serum pool (1:10 dilution, n = 6) was preincubated with serial dilutions of competitors [melon extract, CJP extract as a positive control, and ovalbumin (OVA, purchased from Sigma) as a negative control] at room temperature for 2 hours. The sera were then applied onto a previously prepared n-CPA9-coated microtiter plate for testing IgE cross-reactivity. Subsequent secondary antibody staining and signal detection was carried out as described above.

## Results and discussion

### Molecular Cloning of a New *C. japonica *Pollen Allergen Structurally Belonging to Plant Subtilisin-Like Serine Protease Family

Our comprehensive 2-D IgE immunoblotting study provided a compelling evidence for the presence of 31 unidentified major allergen spots that showed a higher IgE-binding frequency than that of Cry j 2 (40%) [6]. Among these spots, we focused on the spot No. 9 (50% IgE-binding frequency), because its internal amino acid sequence (GHGTHTSSTAA, determined by TOF-MS and tandem MS/MS analyses) was highly homologous to peptidases. Using this sequence information, we cloned full-length cDNA encoding CPA9 by 5'- and 3'-RACE-PCR. Nucleotide sequencing and BLAST analyses revealed that *cpa9 *cDNA composed of 2562 nucleotides encoding 757 amino acids, which was homologous to subtilisin-like serine protease family (data not shown). Its calculated molecular weight and isoelectric point were 80148.79 Da and 6.22, respectively. The CPA9 sequence contained the catalytic triad (D, H, S) responsible for the proteolytic activity of subtilisin-like serine proteases [25]. The phylogenetic analysis of CPA9 polypeptide with other plant serine proteases using ClastalW software showed that CPA9 was a member of clan SB (data not shown). The characteristic amino acid sequences around the catalytic triad also strongly suggested that CPA9 was classified into a member of subtilisin-like peptidase (subfamily S8A), as designated by the MEROPS peptidase database [26]. Intriguingly, we also found that this family contained the melon major allergen Cuc m 1, [27] which was highly homologous to CPA9 (40.1% identity and 55.1% similarity).

### Preparation of r-CPA9 and Specific Antisera

We next tried to produce r-CPA9 using *E. coli *cold shock expression system. SDS-PAGE and anti-His immunoblotting analyses revealed a successful soluble expression of 145 kDa r-CPA9-TF fusion protein. We purified this fusion protein using His-Trap chelate affinity chromatography, and nonfusion r-CPA9 (seen as a single band protein under silver staining of a SDS-PAGE gel) was prepared by thrombin digestion and further purification by nonreducing SDS-PAGE and gel extraction procedures (data not shown). We also prepared murine CPA9-specific antisera upon immunization with the purified r-CPA9.

### Purification of n-CPA9

For further immunochemical characterization of CPA9, we next sought to obtain n-CPA9 from *C. japonica *pollen extract. Two successive chromatographic steps on a HiTrap Butyl HP hydrophobic interaction column and a Mono Q anion exchange column yielded a single 90 kDa protein as seen under reducing SDS-PAGE analysis (data not shown). Immunoblotting also indicated that the obtained 90 kDa protein was recognized by anti-CPA9 polyclonal antibody. Furthermore, TOF-MS/MS analysis demonstrated that 2 internal amino acid sequences of the 90 kDa antigen were completely coincided with those deduced from *cpa9 *cDNA. Taken together, we concluded that the purified 90 kDa molecule was n-CPA9.

### Potent IgE-Binding Capacity of n-CPA9 and Its Cross-Reactivity with Melon Extract

To examine IgE-binding frequency of n-CPA9, we next performed ELISA using sera from 26 Japanese cedar pollinosis patients (RAST score ≥2) and 5 healthy donors as a control. We found that n-CPA9 showed 88.5% IgE binding frequency (23/26), which was higher than that observed against major allergen Cry j 1 (65.4%, 17/26, data not shown). This suggests that CPA9 is an additional major Japanese cedar pollen allergen.

**Figure 1:**
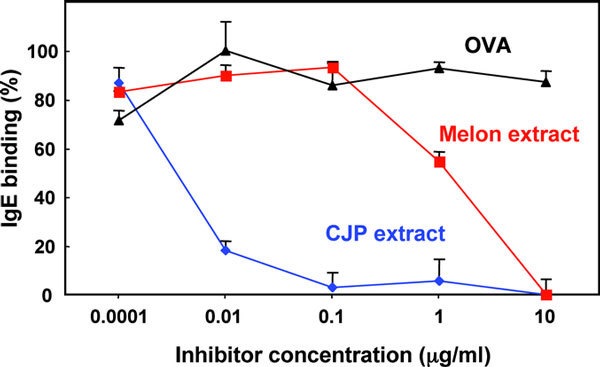
**IgE cross-reactivity of n-CPA9 with melon extract**. Competitive ELISA was performed by preincubation of a pool of CPA9-positive Japanese cedar pollinosis patient sera (n = 6) with serial concentrations of melon extract, CJP extract (positive control), or OVA (negative control).

Because CPA9 polypeptide was highly homologous to the melon subtilisin-like endopeptidase allergen Cuc m 1, [27] we next tested whether r-CPA9 showed IgE cross-reactivity with melon extract. As shown in Figure 1, competitive ELISA revealed that the melon extract inhibited the binding of IgE in pooled patients' sera to immobilized n-CPA9 in a dose-dependent manner; 45% inhibition of IgE-binding was observed upon supplementation of 1 *μ*g/ml melon extract, and preincubation with 10 *μ*g/ml extract completely inhibited IgE-binding. We also found that *C. japonica *pollen extract (CJP extract, positive control) showed a nice inhibitory action, whereas no competition was observed upon incubation with OVA (negative control). These results raise a possibility that this subtilisin-like serine protease might be a new class of plant cross-reactive allergen, although actual IgE-cross-reactivity to Cuc m 1 and to other subtilisin-like allergens needs to be confirmed in future studies.

## Conclusions

In summary, we identified a novel class of Japanese cedar pollen allergen (CPA9) homologous to the plant subtilisin-like serine proteases. Its higher IgE-binding frequency than that of the major allergen Cry j 1 suggests that this molecule should be an additional important allergen in Japanese cedar pollinosis, although further analyses are needed to better characterize its allergenicity and proallergic potency. We believe that CPA9 would be applicable for the development of component-resolved diagnosis and specific immunotherapy of Japanese cedar pollinosis. Furthermore, this molecule offers a good model to assess a possible role of serine protease activity in the pathogenesis of cedar pollinosis.

## End Notes

Ahmed Ragaa Nour Ibrahim and Seiji Kawamoto contributed equally to the work.
